# *c*-axis preferential orientation of hydroxyapatite accounts for the high wear resistance of the teeth of black carp (*Mylopharyngodon piceus*)

**DOI:** 10.1038/srep23509

**Published:** 2016-03-22

**Authors:** Jimin Fu, Chong He, Biao Xia, Yan Li, Qiong Feng, Qifang Yin, Xinghua Shi, Xue Feng, Hongtao Wang, Haimin Yao

**Affiliations:** 1 Department of Mechanical Engineering, the Hong Kong Polytechnic University, Hung Hom, Kowloon, Hong Kong SAR, China; 2 AML, Department of Engineering Mechanics and Center for Mechanics and Materials, Tsinghua University, Beijing 100084, China; 3 Institute of Applied Mechanics, Zhejiang University, Hangzhou, Zhejiang 310027, China; 4 The State Key Laboratory of Nonlinear Mechanics, Institute of Mechanics, Chinese Academy of Sciences, Beijing 100190, China

## Abstract

Biological armors such as mollusk shells have long been recognized and studied for their values in inspiring novel designs of engineering materials with higher toughness and strength. However, no material is invincible and biological armors also have their rivals. In this paper, our attention is focused on the teeth of black carp (*Mylopharyngodon piceus*) which is a predator of shelled mollusks like snails and mussels. Nanoscratching test on the enameloid, the outermost layer of the teeth, indicates that the natural occlusal surface (OS) has much higher wear resistance compared to the other sections. Subsequent X-ray diffraction analysis reveals that the hydroxyapatite (HAp) crystallites in the vicinity of OS possess *c*-axis preferential orientation. The superior wear resistance of black carp teeth is attributed to the *c*-axis preferential orientation of HAp near the OS since the (001) surface of HAp crystal, which is perpendicular to the *c*-axis, exhibits much better wear resistance compared to the other surfaces as demonstrated by the molecular dynamics simulation. Our results not only shed light on the origin of the good wear resistance exhibited by the black carp teeth but are of great value to the design of engineering materials with better abrasion resistance.

In nature, organisms through millions of years of evolution have developed a variety of protective or invasive apparatuses to enhance their survival chance in the severe biological competitions. For example, the mineralized shells of mollusks provide adequate protection to their vulnerable bodies, while the teeth of vertebrates facilitate their predatory activities^1^,^2^. In the recent decades, much effort has been invested in the studies on the protective bioarmors such as sea shells^3^,^4^ and fish scales^5^,^6^, inspiring the developments of novel structural materials with improved mechanical properties. For example, by mimicking the brick-and-mortar structure of nacre in sea shells, people fabricated a variety of composites^7^,^8^,^9^ exhibiting both high toughness and high strength. In contrast to the efforts invested on the bioarmors, relatively less attention has been paid to their invasive rivals^2^,^10^ even though they have comparable value for the development of bioinspired materials as Mother Nature has no bias between prey and predator.

Among diverse invasive biological materials in nature, the pharyngeal teeth of *Mylopharyngodon piceus* (also called black carp) attract our attention due to their unique functionality. Unlike the other fresh-water fishes which are normally herbivorous, black carp mainly feeds on mollusks such as snails and mussels by crushing their protective shells with its powerful pharyngeal teeth as shown in Fig. 1a. Surprisingly, previous study indicated that the black carp teeth exhibit comparable stiffness and hardness as those of mollusk shells. The reason why black carp teeth can crush mollusk shells with comparable mechanical properties has been unraveled from the perspective of structure^11^. More interestingly, like other biological materials the black carp teeth were found to have inclination to ring cracking rather than radial cracking under indentation. Such inclination to cracking mode with less destructibility is not a coincidence but a designed consequence as we revealed recently^12^,^13^. To attain a more comprehensive understanding of the ingenious material design strategies adopted by the black carp teeth, our study is extended further to wear resistance in view of the fact that black carp teeth can function well upon frequent mechanical interactions (e.g. squeezing and grinding) with the hard mollusk shells. Our main goal is to uncover the structural basis accounting for such good wear resistance of black carp teeth, which may provide a novel way to improve the wear resistance of the current synthetic materials particularly those with HAp being the major composition.

## Results

### Nanoscratching tests on the enameloid

**Figure 1:**
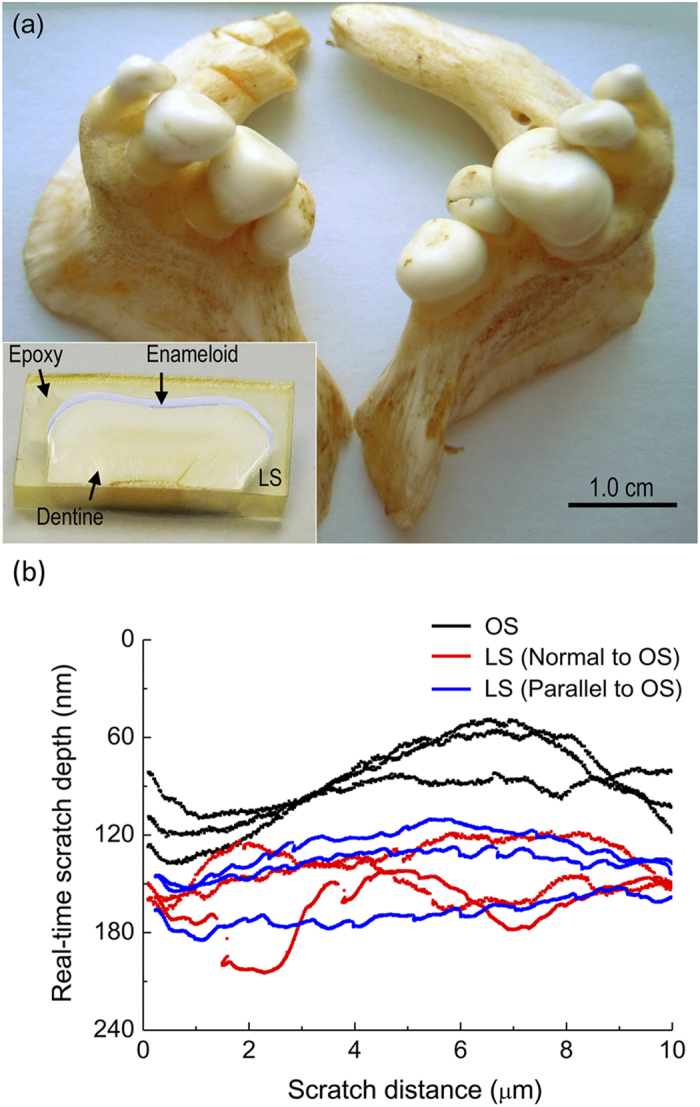
(**a**) Photo of pharyngeal teeth of black carp with inset showing the longitudinal section (LS) of a tooth embedded in epoxy. (**b**)Real-time scratch depths of nanoscratching tests conducted on the OS and LS of enameloid by using Berkovich probe with tip radius around 146 nm and normal load of 2 mN. OS: Occlusal Surface; LS: Longitudinal Section.

**Figure 2:**
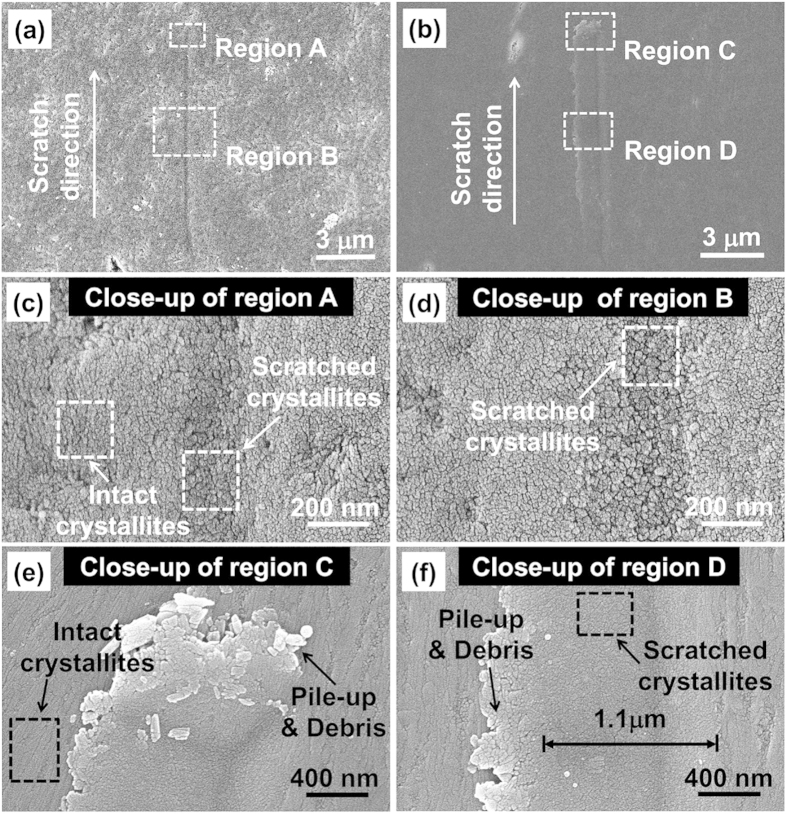
SEM images of the scratches produced by a Berkovich probe on (**a**) OS and (**b**) LS; (**c,d**) close-ups of the regions A and B shown in (**a**); (**e,f**) close-ups of the regions C and D shown in (**b**).

Unlike in the human teeth, the outermost layer of black carp teeth does not possess the characteristic rod structure of enamel and therefore is called enameloid (i.e., resembling enamel)^11^. To quantify the wear resistance of black carp teeth, nanoscratching tests were carried out on the occlusal surface (OS) and longitudinal section (LS) of enameloid respectively for comparison (See Methods for details). The real-time depths of the representative scratches produced by a Berkovich probe are shown in Fig. 1b. It can be seen that the mean depth of the scratch grooves on OS is around 80 nm while that on LS reaches 150 nm irrespective of the scratching direction. Field Emission Scanning Electron Microscopy (FESEM) imaging on the scratched surfaces (See Methods) shows that the response of the OS to scratching differs from that of the LS not only in the size of the residual scratch but also in the failure mode (see Fig. 2a,b). The mean width of the scratch grooves produced on the OS is around 0.2 μm, while that on the LS reaches up to 1.1 μm. Scratching only causes local plastic deformation on the OS (see Fig. 2c,d), whereas it produces obvious ‘pile-up’ and debris on LS (see Fig. 2e,f). The former failure mode is called ‘rubbing’ mode and the latter one is called ‘cutting’ mode. Moreover, before scratching the LS exhibits elongated prismatic texture with longitudinal axis perpendicular to the OS (Fig. 2e,f)^11^. Such prismatic structure is crushed into granules with diameter around tens of nanometers after scratching, as displayed by Fig. 2e,f. In contrast, the ‘granular’ texture exhibited by the OS is preserved very well after scratching, as shown in Fig. 2c,d. Such distinct responses of OS and LS to scratching imply that the OS of the black carp teeth possesses much higher wear resistance compared to the LS. This result also holds for the scratching with probes of different shapes and sizes (see Supplementary Information).

### Comparative XRD analysis of enameloid

**Figure 3:**
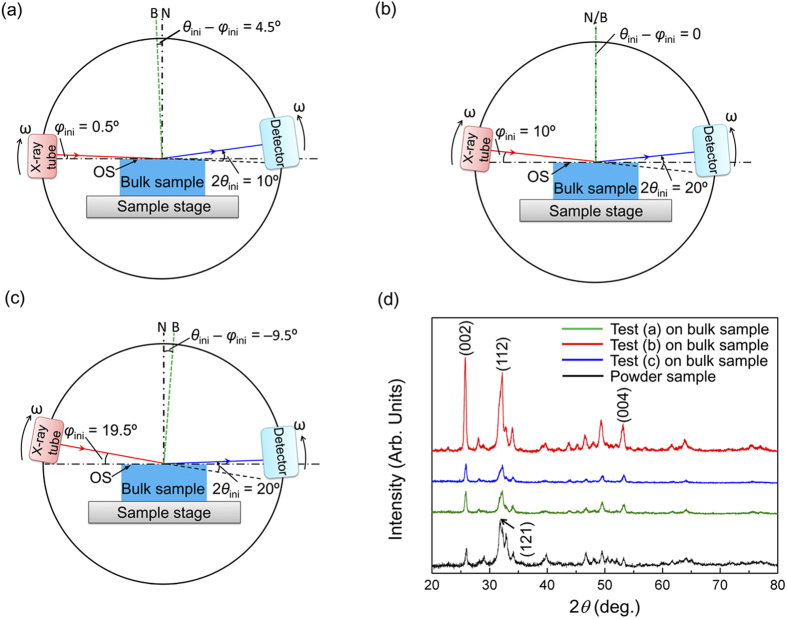
(**a–c**) Schematics of the configurations of three XRD tests on bulk enameloid sample: (**a**) *φ*_ini_ = 0.5°, 2*θ*_ini_ = 10°; (**b**) *φ*_ini_ = 10°, 2*θ*_ini_ = 20°; (**c**) *φ*_ini_ = 19.5°, 2*θ*_ini_ = 20°. *φ*_ini_: Initial incident angle; 2*θ*_ini_: initial diffraction angle; *ω*: scanning angular speed; **N**: normal of sample surface; **B**: Diffraction vector (vector that bisects of the angle between the incident and diffracted beam). (**d**) Diffraction patterns of three XRD tests on bulk sample in comparison with that obtained from powder sample.

To reveal the structural origin accounting for the prominent wear resistance of OS as discovered above, comparative X-ray diffraction (XRD) analysis was carried out. Traditional XRD analysis usually uses powder specimen, so that diffraction beams reflected from various crystallographic planes can be detected due to the random orientation of grains in powder specimen. If a bulk material, in which the layout of building crystallites is not as random as in powder, is used directly as the XRD specimen, the obtained diffraction pattern may deviate from that of its powder. Specifically, the signal intensity of the beam diffracted by the crystallographic planes with preferential alignment would be enhanced to the greatest extent when the diffraction vector (i.e., the vector that bisects the angle between the incident and reflected beams) coincides with the normal of these planes. Inspired by this speculation, we conducted a comparative XRD analysis on bulk and powder specimens of the enameloid to verify the existence of preferential orientation (See Methods for details). For the bulk specimen, three tests with different initial incident angles, *φ*_ini_ and initial diffraction angles, 2*θ*_ini_, were carried out, as schematically illustrated by Fig. 3a–c. As the diffractometer we used was operated in the Bragg-Brentano *θ–θ* configuration, in which the X-ray tube and detector rotate simultaneously at the same angular speed *ω* but in opposite directions during scanning, the diffraction vector, **B**, is unchanged during the test. As a consequence, if the initial incident angle is chosen as *φ*_ini_ = *θ*_ini_, the diffraction vector **B** would coincide with the normal of the sample surface (or the OS for our bulk enameloid sample), as shown in Fig. 3b. Otherwise, if *φ*_ini_ ≠ *θ*_ini_, the diffraction vector **B** would deviate from the normal of OS, **N**, by an angle of *φ*_ini_–*θ*_ini_, as shown in Fig. 3a,c.

Figure 3d shows the diffraction patterns of three XRD tests on a bulk enameloid sample compared to that of the powder specimen made from the bulk enameloid by grinding. As expected, all patterns exhibit the characteristic XRD peaks of hydroxyapatite (HAp), confirming that the major composition of enameloid is HAp. However, the relative intensities of the characteristic peaks differ among the patterns. In the pattern obtained from the powder specimen, the highest intensity is obtained from the diffraction by the (121) planes at 2*θ* = 31.77° and reaches about 2 times of the intensity diffracted by the (002) planes at 2*θ* = 25.8°. In contrast, in the patterns obtained from the bulk specimen, the intensity corresponding to the diffraction by the (002) planes gets enhanced to different extents. Comparison between the patterns of cases (a), (b) and (c) shows that such enhancement gets maximized in case (b), giving rise to a pattern in which the intensity corresponding to the (002) planes is predominantly high and reaches 1.2 times of that by the (112) planes at 2*θ* = 32.1°. The relatively higher intensity for (002) planes in case (b) implies that more (002) planes involve in reflecting the incident beam in case (b) compared to the cases (a) and (c). That is, in case (b) most of the HAp crystallites near the OS have (002) planes perpendicular to the diffraction vector **B**, which coincides with the normal of sample surface or OS. In view of the hexagonal close packed (HCP) crystallographic structure of HAp in which *c*-axis is perpendicular to the (002) planes, above XRD results suggest that a significant portion of HAp crystallites near the OS have *c*-axises normal to the OS. The *c*-axis preferential orientation near the enameloid surface is thus confirmed.

### Molecular dynamics simulation of nanoscratching on single crystal HAp

**Figure 4:**
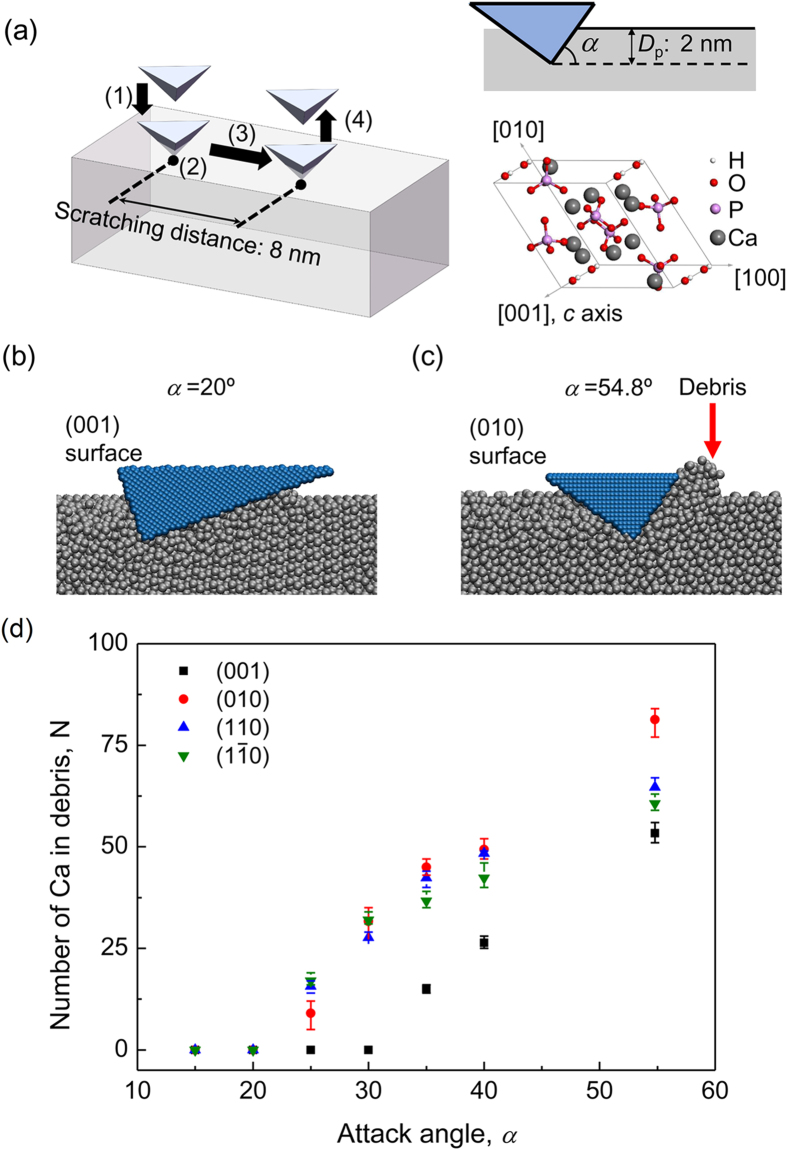
(**a**) Schematics of the MD simulation model and unit cell of HAp crystal. The virtual nanoscratching test is implemented through four consecutive steps: (1) the rigid probe engages with the HAp single crystal by penetrating to depth *D*_p_; (2) the probe holds still for 20 ps for relaxation; (3) the probe is displaced horizontally to scratch the HAp by 8 nm with *D*_p_ = 2 nm kept constant, and (4) the probe is withdrawn from the HAp sample. (**b,c**) Snapshots of scratching on (001) and (010) surfaces at the end of step (3) with corresponding attack angle *α* being 20° and 54.8° respectively. Here only the Ca atoms in HAp are shown for a clearer visualization. (**d**) Variations of the amount of debris characterized by the number of Ca atoms scratched away from the bulk HAp as a function of attack angle *α*. The error bar on each data point was based on the statistics of the results along three different directions on a given surface.

Having shown the superior wear resistance of the OS of black carp teeth as well as the *c*-axis preferential orientation of the building HAp crystallites near the OS, seeking the possible intrinsic correlation between them naturally becomes our follow-up objective. For this purpose, molecular dynamics (MD) simulation was applied to investigate the response of single crystal HAp to scratching at atomic length scale (See Methods for details). Figure 4a shows the simulation model, in which a rigid pyramidal probe is initially compressed on a single crystal HAp to depth *D*_p_ = 2 nm and then displaced horizontally at a constant speed and constant attack angle *α* (i.e., the angle between the scratching surface of the probe and the horizontal plane). To quantify the extent of abrasion, the amount of debris was computed by counting the number of Ca atoms scratched away from the bulk HAp^14^,^15^,^16^,^17^,^18^,^19^,^20^,^21^. A Ca atom is counted as debris if its displacement exceeds a critical value of 1.0 nm, which is roughly the HAp lattice constant or the cutoff distance of the force field^14^,^15^,^16^,^17^,^18^. We simulated the scratching tests on four representative surfaces of a single crystal HAp respectively including (001), (010), (110), and 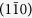
surfaces with attack angle *α* varying from 15° to 54.8°. On each surface, scratching simulations were conducted along three different directions as shown in Table 1 to examine the possible directional dependence. While the (001) surface is parallel to the preferentially orientated surface (002) and therefore OS, the other three surfaces are of particular interest because they are perpendicular to (001) and thus may be assumed by the LS. Due to the structural symmetry of HAp crystal, the (100) surface should have the same behavior as that of the (010) surface and therefore is not considered individually^22^,^23^,^24^.

Our simulation results showed that the behavior of HAp single crystal under scratching depends on the attack angle *α*. At smaller attack angle, surfaces under scratching exhibit ‘rubbing mode’ failure (Fig. 4b), while at lager attack angle *α* ‘cutting mode’ failure occurs (Fig. 4c). There exists a critical attack angle, *α*_c_, above which ‘cutting mode’ dominates or, in other words, debris is produced. Figure 4d shows the variation of the amount of debris, characterized by the number of Ca atoms scratched off from the HAp, as a function of attack angle. It can be seen that *α*_c_ is around 30° for the (001) surface, while for the other surfaces it is around 20°. Considering the fact that the black carp teeth in service may experience attacks from different angles, larger *α*_c_ exhibited by the (001) surface implies that the (001) surface has higher probability, in comparison to the other surfaces, to give rise to ‘rubbing mode’ failure under attacks with random attack angles. This may also explain why in our scratching experiments shown in Fig. 2 ‘rubbing’ mode was observed on the OS while ‘cutting’ mode on LS. On the other hand, from the perspective of abrasion, ‘cutting mode’ failure is more destructive than the ‘rubbing mode’ one as it involves the removal of material in addition to the plastic deformation. It can be observed from Fig. 4d that the amount of debris produced on (001) surface of HAp is always less than the debris produced on the other surfaces at the same attack angle, implying the higher wear resistance of the (001) surface. Recalling the *c*-axis preferential orientation of the HAp crystallites near the OS as revealed by XRD analysis, the superior wear resistance of black carp teeth can be attributed to the *c*-axis preferential orientation of the HAp crystallites near the OS.

### Theoretical modeling

To gain a deeper understanding of the distinct behavior of the (001) surface from the other counterparts under scratching, theoretical analysis was carried out. From the energy point of view, material under scratching tends to assume failure mode requiring less energy or more energetically preferable. For a constant-speed scratching on a generic solid surface, the energy required for unit scratching distance equals the horizontal driving force *F* applied on the probe, which is given by^25^,^26^


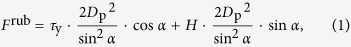



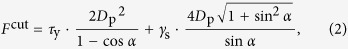


for ‘rubbing mode’ and ‘cutting mode’ failure, respectively (See Supplementary Information for the detailed derivation). In Eqs (1) and (2), *τ*_y_, *H* and *γ*_s_ are the shear strength, hardness and surface energy of the scratched material respectively; *α* is the attack angle and *D*_p_ is the penetration depth. Although Eqs (1) and (2) were developed for isotropic solids^25^,^26^, we assume they also apply to anisotropic materials as long as the mechanical properties (*H, τ*_y_ and *γ*_s_) are taken as the effective values obtained from the surfaces under scratching. The failure mode under scratching is determined by the competition between *F*^ rub^ and *F*^ cut^. If *F*^ rub^ < *F*^ cut^, ‘rubbing mode’ happens; otherwise, ‘cutting mode’ takes place. At the critical attack angle, *α*_c_, we have *F*^ rub^ = *F*^ cut^, which implies


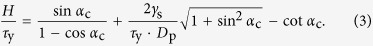


**Figure 5:**
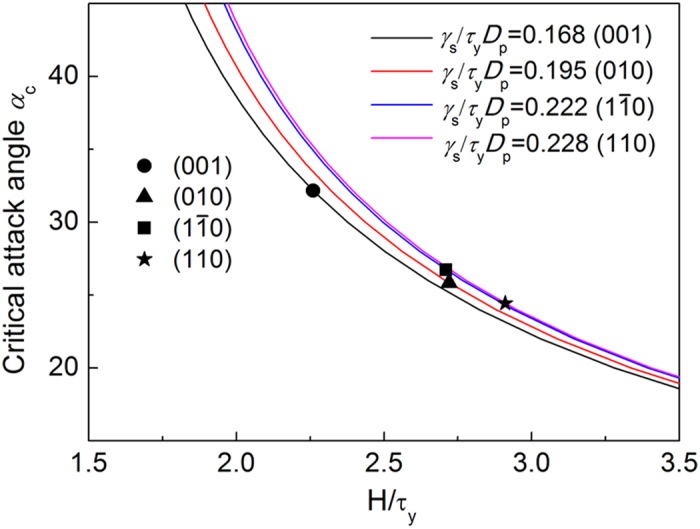
Dependence of *α*_c_ on *H*/*τ*_y_ for given *γ*_s_/*τ*_y_*D*_p_.

Equation 3 implicitly defines the critical attack angle, *α*_c_, as a function of two dimensionless parameters 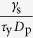 and 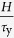. Taking penetration depth *D*_p_ = 2 nm, the critical attack angle for surfaces of (001), (010), (110), and 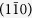 of single crystal HAp can be determined graphically based on their respective effective *H, τ*_y_ and *γ*_s_ (Table 2) calculated by MD simulation (See Supplementary Information for details). As shown by Fig. 5, *α*_c_ ≈ 25° for (010), (110) and 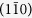, while *α*_c_ ≈ 33° for (001), agreeing well with our MD simulation results shown in Fig. 4d. The relatively higher *α*_c_ for (001), according to Fig. 5, is mainly due to its relatively lower ratio of *H*/*τ*_y_.

It should be pointed out that our MD simulation as well as above theoretical analysis implies that ‘rubbing mode’ failure can also be produced on the surfaces other than OS, which has not been observed in the earlier scratching experiment with Berkovich probe. We therefore repeated the scratching tests using a blunt conical probe so as to achieve a much smaller attack angle. As expected, ‘rubbing mode’ failure was observed not only on OS but also on LS (See Supplementary Information for details).

## Discussion

In summary, inspired by the outstanding wear resistance exhibited by the OS of black carp teeth, we carried out a comparative XRD analysis on its enameloid and uncovered the *c*-axis preferential orientation of the building HAp crystallites near the OS. Subsequent MD simulation demonstrated that the (001) surface of single crystal HAp exhibits better wear resistance compared to other surfaces. Thus, the dependence of the outstanding wear resistance of black carp teeth on the *c*-axis preferential orientation of HAp was confirmed. Similar preferential crystallographic orientation was also observed in the other natural materials such as bones^27^, oyster shell^28^,^29^, and tooth of sea urchin^30^. To further reveal the mechanics accounting for the orientation-dependent wear resistance of HAp crystal, theoretical analysis was performed. It was revealed that the (001) surface of HAp, compared to the other crystallographic surfaces, is more prone to have plastic deformation (‘rubbing mode’ failure) rather than removal of materials (‘cutting mode’ failure) under scratching, giving rise to its higher wear resistance. In biomedical field, HAp-based materials have been widely used as implant materials and fillers for repairing bone and teeth^31^ due to the excellent biocompatibility of HAp. But the synthetic HAp normally does not have good mechanical properties^32^,^33^, which restricts its applications in load-bearing materials such as coating of artificial joint and crown of synthetic teeth. The finding of our work implies great promise of applying preferential crystallographic orientation to enhance the wear resistance of these HAp-based biomedical materials even though the technique to align the crystals of HAp remains a challenge.

## Methods

### Nanoscratching test

The longitudinal section (LS) was obtained by slicing a fresh black carp tooth perpendicularly to the occlusal surface (OS) using a diamond saw (Minitom, Struers). With lubrication of DI water, samples were polished on the occlusal surface (OS) or longitudinal section (LS) with 2000 and 4000 grit sand papers (Silicon Carbide Waterproof Paper, PROFIT) for 5 min, followed by lapping with cloth pad (Micropad Extra, Pace Technologies) in combination with 0.6 μm silica colloid solution (Pace Technologies). Subsequently, samples were rinsed with DI water thoroughly and dried in air. Atomic force microscopy confirmed that the height of roughness asperities of the polished surfaces ranged from a few nanometers to tens of nanometers, depending on the section orientation. Nanoscratching tests (Triboindenter 950) were conducted on both OS and LS of enameloid using diamond Berkovich probe (TI-0039, tip radius = 146 nm). Scratching tests on the LS were conducted along directions parallel and normal (outwards) to the OS, respectively. To reduce the effect of the mechanical inhomogeneity on the tests, all the experiments on the LS were conducted on the areas very close (<40 μm) to the OS, in which the variations of modulus and hardness are negligibly small. No particular direction was selected for the scratching on the OS. To verify the repeatability of the results, scratching along a specific direction was repeated for three times. To avoid the interference between adjacent scratches, the inter-scratch spacing was taken as 10 μm, which was about 9 times of the largest scratch width. The normal load was constantly taken as 2 mN. The scratching distance and scratching speed were taken as 10 μm and 0.5 μm/s, respectively. Scratched samples were sputtered with gold in vacuum and FESEM (S-4800, Hitachi) imaging was conducted with accelerating voltage of 5 kV. To examine the effects of probe size and shape, similar scratching tests were also conducted using a 90° conical probe (TI-0041, tip radius = 20 μm) with normal force being 10 mN. Related results are shown in Fig. S6.

### X-ray diffraction

X-ray diffraction was conducted on enameloid specimens in both powder and bulk forms. The powder specimen was made from the bulk ones by grinding. For the bulk specimen, three XRD tests, denoted by cases (a), (b) and (c), were conducted on the OS by taking the initial incident angle *φ*_ini_ = 0.5°, 10°, 19.5° and initial diffraction angle 2*θ*_ini_ = 10°, 20°, 20° respectively, as shown in Fig. 3a–c. So that, in cases (a), (b) and (c) the diffraction vector, **B**, deviated from the normal of sample surface, **N**, by 4.5°, 0°, *−*9.5° respectively. The bottom side of the bulk specimen (i.e. the opposite side of the OS) was polished well before being mounted onto the sample stage so as to ensure that the OS was parallel to the sample stage surface. All the X-ray diffraction (XRD) tests were carried out using an X-ray diffractometer (SmartLab, Rigaku) with Cu*k*_*α*_radiation of 1.5418 Å wavelength working at 45 kV and 200 mA and scan speed of *ω* = 0.08°s^−1^ in the Bragg-Brentano *θ*–*θ* configuration. The scan range was taken as 2*θ* = 10°~80°, 20°~90°, 20°~80° in cases (a), (b) and (c), respectively. The scan range for the test on the powder specimen was taken as 2*θ* = 10° ~ 80°. All the diffraction patterns obtained were compared with the standard pattern for HAp (International Centre of Diffraction Data, Powder Diffraction File, No: 84–1998).

### Molecular dynamics simulation

The MD simulations of nanoscratching were performed with the LAMMPS package^34^. The extended CHARMM (Chemistry at Harvard Macromolecular Mechanics) force field was used to describe the interactions between atoms in the HAp crystals^22^,^23^. The CHARMM force field was used to compute the interatomic Lennard-Jones and Coulombic interactions with an additional switching function that ramps the energy and force smoothly to zero between inner and outer cutoffs^34^,^35^. In our simulations, the inner and outer cutoffs were chosen as 6 Å and 10 Å, respectively, so that all atoms inside a HAp unit cell could interact with each other. Four simulation samples were generated: in the first sample the upper surface to be scratched was (001) plane (see Fig. 4b), while in the other samples the upper surfaces to be scratched were (010) (see Fig. 4c), (110), and 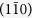 planes. The atoms at the bottom surface in each sample were fixed in all dimensions. To eliminate edge effect, each sample contained more than 60 unit cells in each dimension with periodical boundary conditions (PBC) applied on all boundaries except the top and bottom surfaces. Each sample was initially relaxed using the canonical ensemble (NVT) for 200 ps. The time step of the simulations was taken as 1 fs and temperature was controlled at 300 K with the Langevin thermostat. Visualization program Visual Molecular Dynamics (VMD)^36^ was used to visualize and output the simulations results. The pyramidal probe (cube-corner) was defined as rigid, consisting of close-packed atoms. Any substantial atom contacting with the probe surface would experience a repulsive force obeying the Lennard-Jones potential 
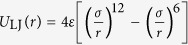
 with a cutoff being 2.2 nm, where ε was taken as 0.01 kcal/mole, σ was taken as 2.0 nm and *r* was the distance from the substantial atom to the atom of the probe. For each surface, scratching simulations were conducted along three different directions, as shown in Table 1.

## Additional Information

**How to cite this article**: Fu, J. *et al*. c-axis preferential orientation of hydroxyapatite accounts for the high wear resistance of the teeth of black carp (*Mylopharyngodon piceus*). *Sci. Rep.* **6**, 23509; doi: 10.1038/srep23509 (2016).

## Supplementary Material

Supplementary Information

## Figures and Tables

**Table 1:** Scratching directions adopted in MD simulations.

**Planes**	**Scratching directions**
(001)	[100], [110] and 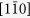
(010)	[100], [001] and [101]
(110)	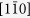 , 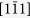 and 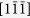
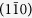	[110], [111] and 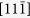

**Table 2:** The values of *H/*τ_y_ and *γ*_s_ */*τ_y_ *D*_p_ calculated from the (001), (010), (110), and (1 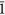 0) planes of single crystal HAp.

**Planes**	***H/τ***_**y**_	***γ***_**s**_***/τ***_**y**_***D***_**p**_
(001)	2.26	0.168
(010)	2.72	0.195
(110)	2.91	0.228
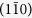	2.71	0.222

*D*_p_ is taken as 2 nm.
