# Supplementary material for: c-axis preferential orientation of hydroxyapatite accounts for the high wear resistance of the teeth of black carp (Mylopharyngodon piceus)
Source: Sci Rep. 2016 Mar 22;6:23509. doi: 10.1038/srep23509 (PMC4802323; doi:10.1038/srep23509)
Supplement: Supplementary Information [file srep23509-s1.pdf]

Supplementary Information

c-axis preferential orientation of hydroxyapatite accounts for the high wear resistance of the teeth of black carp (*Mylopharyngodon piceus*)

Jimin Fu[1,†], Chong He[1,†], Biao Xia[2], Yan Li[2], Qiong Feng[3], Qifang Yin[1], Xinghua Shi[4], Xue Feng[2], Hongtao Wang[3], and Haimin Yao[1,*]

[1]Department of Mechanical Engineering, the Hong Kong Polytechnic University, Hung Hom, Kowloon, Hong Kong
[2]AML, Department of Engineering Mechanics and Center for Mechanics and Materials, Tsinghua University, Beijing 100084, China
[3]Institute of Applied Mechanics, Zhejiang University, Hangzhou, Zhejiang 310027, China
[4]The State Key Laboratory of Nonlinear Mechanics, Institute of Mechanics, Chinese Academy of Sciences, Beijing 100190, China

[*] Correspondence and requests for materials should be addressed to H.Y. (E-mail: mmhyao@polyu.edu.hk, Tel.: +852 2766 7817)
[†] These authors contributed equally to this work.

Determination of the mechanical properties of HAp by molecular dynamics (MD) simulations

MD simulations were carried out to determine the mechanical properties of HAp using the same force field as in the scratching simulation (See Methods in the main text).

Determination of shear strength τ_y. To estimate the shear strengths of the HAp between (001) planes, (010) planes, (110) planes, and $(1\bar{1}0)$ planes along the [100] direction, [100] direction, $[1\bar{1}0]$ direction and [110] direction, respectively, two-dimensional shear simulations were carried out. As shown in Fig. S1a, the height of each sample is roughly 4 nm. In the simulations, shear strain was applied by changing

the tilting angle β of sample at the strain rate of $10^{10}s^{-1}$. The shear stress-strain curves are shown in Fig. S1 (b-e), from which the shear strengths (the peak stresses on the curves), for (001), (010), (110), and $(1\bar{1}0)$ are obtained (**Table S1**).

Determination of hardness *H*. To determine the hardness of HAp crystal on the (001), (010), (110) and $(1\bar{1}0)$ surfaces, virtual nanoindentation tests were carried out using MD simulation. In each set of the simulations, the HAp sample has more than 60 unit cells in all three dimensions to eliminate edge effect, and the atoms at the bottom surface are fixed in all dimensions. As shown in Fig. S2a, a cube-corner indenter was applied with the maximum indentation depth h_{max} = 1.5 nm. The load-depth curves are shown in Fig. S2 (b-e), where dashed lines represent the fitting curves of the unloading portions to be used to determine the hardness H and the reduced modulus E_r using Oliver-Pharr (O-P) model[1]. The calculated results are shown in Table S1, which is in consistence with those measured by experiments[2,3].

Determination of surface energy γ_s. Surface energy of HAp refers to the energy associated with the creation of new surface of unit area. It can be obtained by calculating the change of total potential energy as the HAp is split into two pieces (the schematics of the simulation procedures is shown in Fig. S3). That is, surface energy $\gamma_s = (E_f - E_i)/A$, where E_f and E_i refer to the total potential energies of the system after and before the fracture, and A stands for the area of newly created surfaces which equals double of the fractured surface area. The calculated surface energies for (001),

(010), (110), and ($1\bar{1}0$) planes are listed in Table S1, which agree with the values reported in literature[4-6].

Theoretical modelling of scratching

To predict the failure mode of surface under scratching, the knowledge of horizontal pushing force F for 'rubbing mode' and 'cutting mode' is necessary. Actually, related results have been obtained especially for scratching with pyramidal probe[1,7-14]. In the theoretical analysis, pile-up or sink-in is neglected. So that the contact area can be determined by the penetration depth D_p and the attack angle α. Additionally, interaction between probe and scratched material is assumed frictionless and non-adhesive.

According to Bowden and Tabor's theory[12], the horizontal pushing force in 'rubbing mode' (as shown in Fig. S4b) F^{rub} was obtained for the pyramidal probe having inclined face in contact with the material during scratching by Sedriks and Mulhearn[13],

$$F^{\mathrm{rub}} = \tau_\mathrm{y} \cdot A^{\mathrm{rub}} \cos\alpha + H \cdot A^{\mathrm{rub}} \sin\alpha \,, \qquad \text{(S1)}$$

where τ_y is the shear strength of the substrate material, H is the indentation hardness, α is the attack angle, and A^{rub} is the actual contact area between the substrate material and the inclined face of the probe. In our model using the cube-corner probe, we have

$$A^{\mathrm{rub}} = \frac{c \cdot D_\mathrm{p}^{\;2}}{\sin^2\alpha}, \qquad \text{(S2)}$$

where c =2 based on Sedriks and Mulhearn's analysis[13,14]. Combination of Equation S1 and S2 gives rise to

$$F^{\mathrm{rub}} = \tau_\mathrm{y} \cdot \frac{2D_\mathrm{p}^2}{\sin^2\alpha} \cdot \cos\alpha + H \cdot \frac{2D_\mathrm{p}^2}{\sin^2\alpha} \cdot \sin\alpha \,. \qquad \text{(S3)}$$

For ‘cutting mode’, Atkins *et al*. established a model (as shown in Fig. S4c)[14] , based on which the horizontal driving force F^{cut} can be given by

$$F^{\mathrm{cut}} = \tau_{\mathrm{y}} \cdot \gamma \cdot A^{\mathrm{cut}} + \gamma_{\mathrm{s}} \cdot A^{\mathrm{new}}, \tag{S4}$$

where γ_{s} is the surface energy and γ is the shear strain of the debris peeled off. The shear strain γ is associated with the inclined angle ϕ of shear plane and attack angle α through[14]

$$\gamma = \sin\alpha / \sin(\alpha - \phi)\sin\phi . \tag{S5}$$

In Equation S5, the inclined angle is determined to be $\phi = \frac{\alpha}{2}$ based on the assumption that the debris happens along the direction requiring the least energy or minimum γ. The cutting plane is the cross-section of the groove, which is normal to the scratch direction, and the area of the cutting plane can be expressed as

$$A^{\mathrm{cut}} = D_{\mathrm{p}}{}^{2} \big/ \sin\alpha . \tag{S6}$$

The area of the new created surface per unit scratching distance can be approximated by

$$A^{\mathrm{new}} \approx 4D_{\mathrm{p}} \cdot \sqrt{1+\sin^{2}\alpha} \big/ \sin\alpha . \tag{S7}$$

So the expression of the horizontal driving force for ‘cutting mode’, F^{cut}, can be written as

$$F^{\mathrm{cut}} = \tau_{\mathrm{y}} \cdot \frac{2D_{\mathrm{p}}{}^{2}}{1-\cos\alpha} + \gamma_{\mathrm{s}} \cdot \frac{4D_{\mathrm{p}}\sqrt{1+\sin^{2}\alpha}}{\sin\alpha} . \tag{S8}$$

Nanoscratching tests by blunt conical probe

Both theoretical analysis and MD simulation indicates that the failure modes of LS of

black carp teeth under scratching may depend on the attack angle α. But in our nanoscratching experiment with Berkovich probe, only ‘cutting mode’ was observed on the LS. To verify the occurrence of ‘rubbing mode’ on LS, nanoscratching tests were repeated by using conical probe with tip radius around 20 μm. Figure S5 compares the geometries of conical probe and the Berkovich probe we applied earlier. Clearly, the conical probe is much blunter than the Berkovich one due to its larger tip radius, implying that the conical probe has much smaller attack angle α. In the nanoscratching with the conical probe, the normal force applied is around 10 mN, resulting in the penetration depth D_{p} around 100 nm and D_{p}/R about 0.005. That is, the equivalent attack angle α, roughly equal to $\cos^{-1}(1-D_{\mathrm{p}}/R)$, is less than 10°. This value is far below the α_{c} of (010), (110), and $(1\bar{1}0)$ surface as predicted by our theoretical modeling. Figure S6 (a-c) shows the atomic force microscope (AFM) images of the residual scratches produced on the LS in comparison with that on the OS. It can be seen that scratching does not produce obvious debris on both LS and OS, and no ‘cutting mode’ failure is observed, agreeing well with the theoretical prediction. However, the depth of the residual scratch on the OS is less than half of that on the LS (see Fig. S6d), implying the higher wear resistance of OS over LS.

References

1 Oliver, W. C. & Pharr, G. M. An improved technique for determining hardness and elastic modulus using load and displacement sensing indentation experiments. *J. Mater. Res.* **7**, 1564-1583, (1992).

2 Saber-Samandari, S. & Gross, K. A. Micromechanical properties of single crystal hydroxyapatite by nanoindentation. *Acta Biomater.* **5**, 2206-2212, (2009).

3 Zamiri, A. & De, S. Mechanical properties of hydroxyapatite single crystals from nanoindentation data. *J. Mech. Behav. Biomed. Mater.* **4**, 146-152, (2011).

4 Chiatti, F., Corno, M. & Ugliengo, P. Stability of the Dipolar (001) Surface of Hydroxyapatite. *J. Phys. Chem. C* **116**, 6108-6114, (2012).

5 Zhu, W. H. & Wu, P. Surface energetics of hydroxyapatite: a DFT study. *Chem. Phys. Lett.* **396**, 38-42, (2004).

6 Corno, M., Busco, C., Bolis, V., Tosoni, S. & Ugliengo, P. Water Adsorption on the Stoichiometric (001) and (010) Surfaces of Hydroxyapatite: A Periodic B3LYP Study. *Langmuir* **25**, 2188-2198, (2009).

7 Johnson, K. L. The correlation of indentation experiments. *J. Mech. Phys. Solids* **18**, 115-126, (1970).

8 Yu, W. P. & Blanchard, J. P. An elastic-plastic indentation model and its solutions. *J. Mater. Res.* **11**, 2358-2367, (1996).

9 Oliver, W. C. & Pharr, G. M. Measurement of hardness and elastic modulus by instrumented indentation: Advances in understanding and refinements to methodology. *J. Mater. Res.* **19**, 3-20, (2004).

10 Bolshakov, A. & Pharr, G. M. Influences of pileup on the measurement of mechanical properties by load and depth sensing indentation techniques. *J. Mater. Res.* **13**, 1049-1058, (1998).

11 McElhaney, K. W., Vlassak, J. J. & Nix, W. D. Determination of indenter tip geometry and indentation contact area for depth-sensing indentation experiments. *J. Mater. Res.* **13**, 1300-1306, (1998).

12 Bowden, F. P. & Tabor, D. *The friction and lubrication of solids*. (Clarendon Press, 1950).

13 Sedriks, A. & Mulhearn, T. Mechanics of cutting and rubbing in simulated abrasive processes. *Wear* **6**, 457-466, (1963).

14 Atkins, A. G. & Liu, J. H. Toughness and the transition between cutting and rubbing in abrasive contacts. *Wear* **262**, 146-159, (2007).

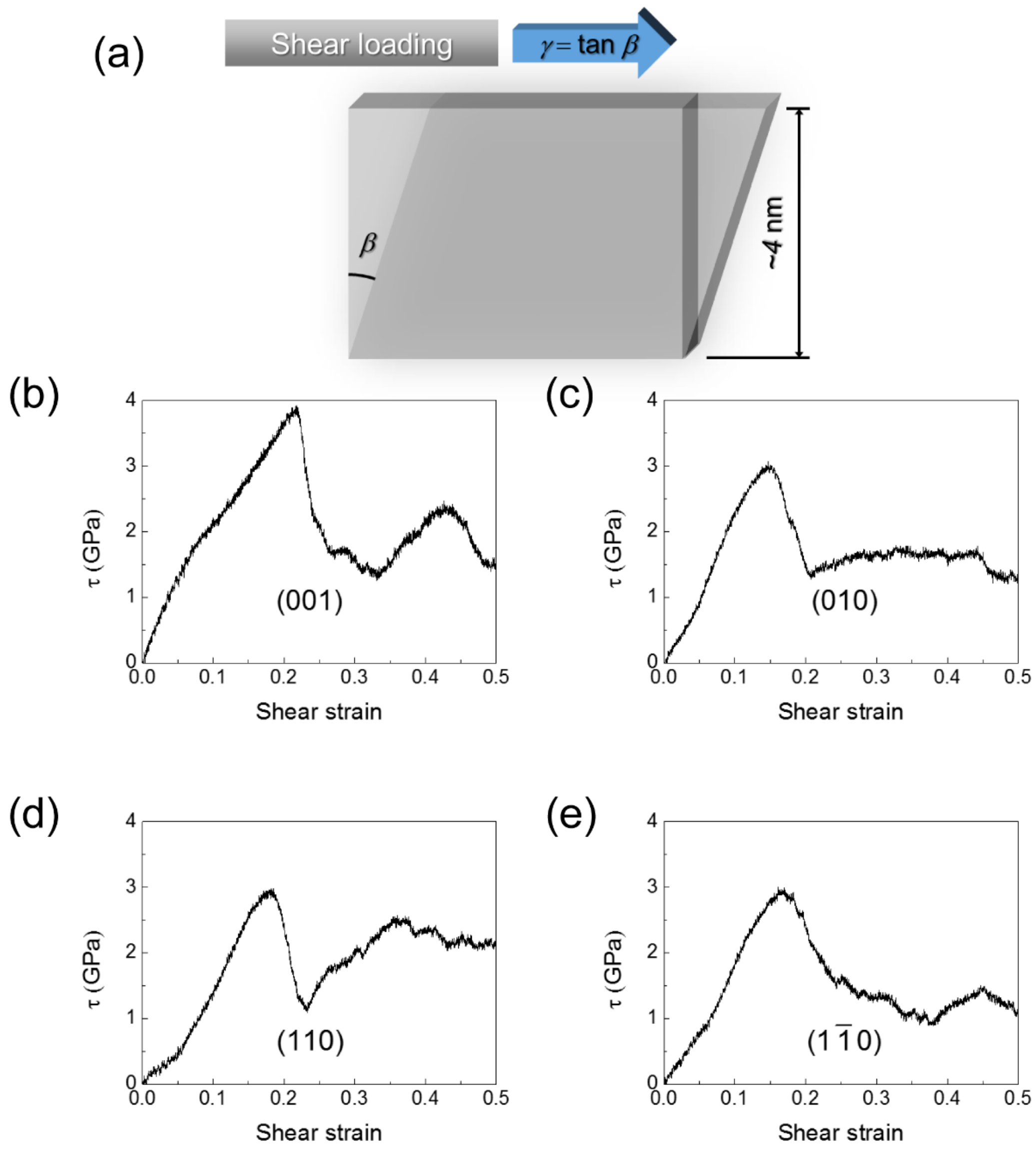

Figure S1. Simple shear simulations. (a) Schematics of the simulations model for shear tests. (b-e) Stress–strain curves of the shear tests.

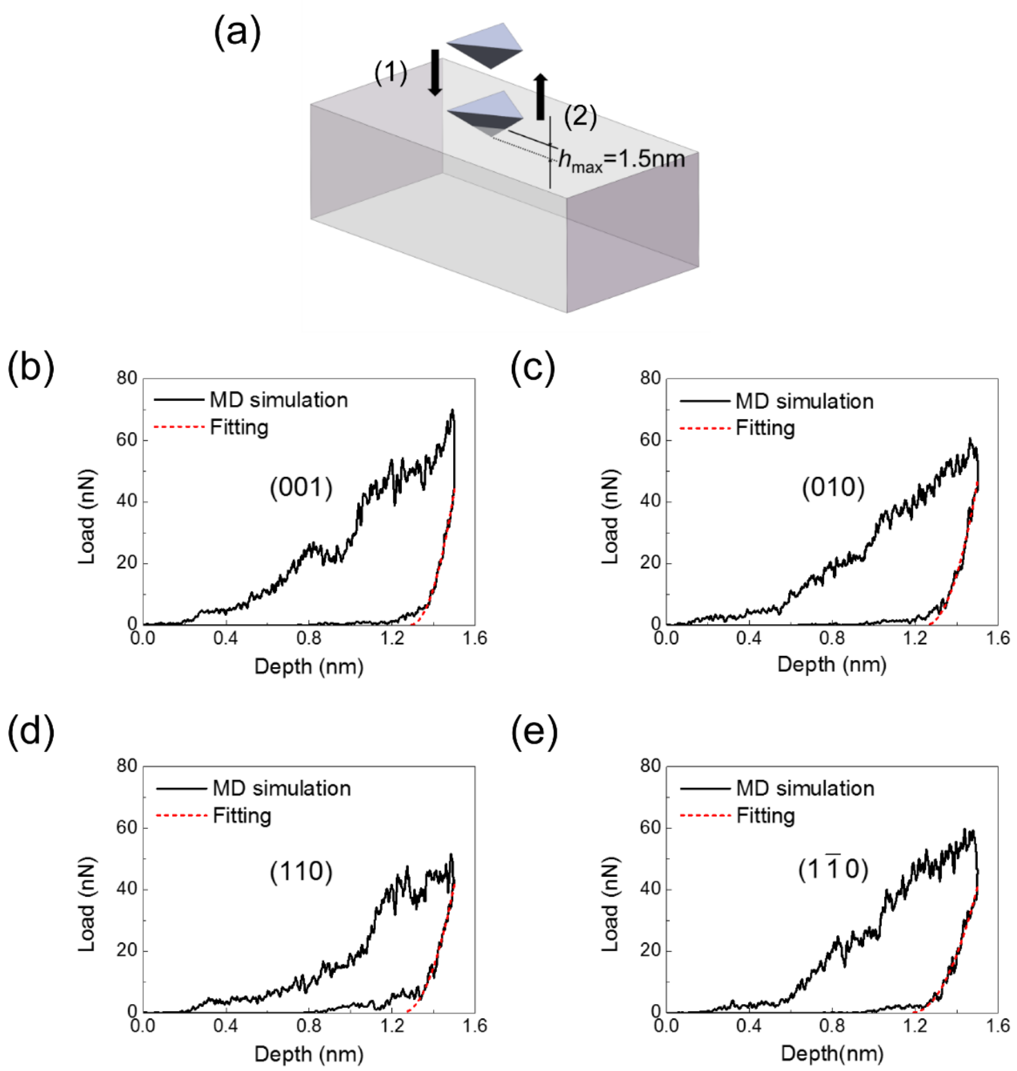

Figure S2. Nanoindentation simulations used to predict the mechanical properties of HAp on different directions. (a) Schematics of the simulations model for indentation tests which are implemented through two consecutive steps: (1) the rigid probe engages with the HAp single crystal with indentation depth *h* =1.5 nm; (2) the probe is withdrawn from the HAp sample. (b-e) Load-depth curves of the indentation simulation obtained on different surfaces, from which the mechanical properties of the related surface can be deduced.

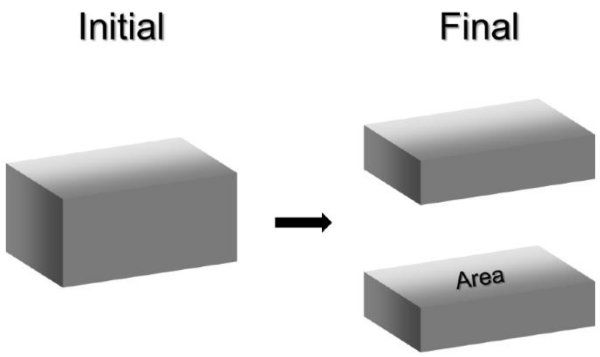

Figure S3. Schematics of the simulations model for determination of surface energy.

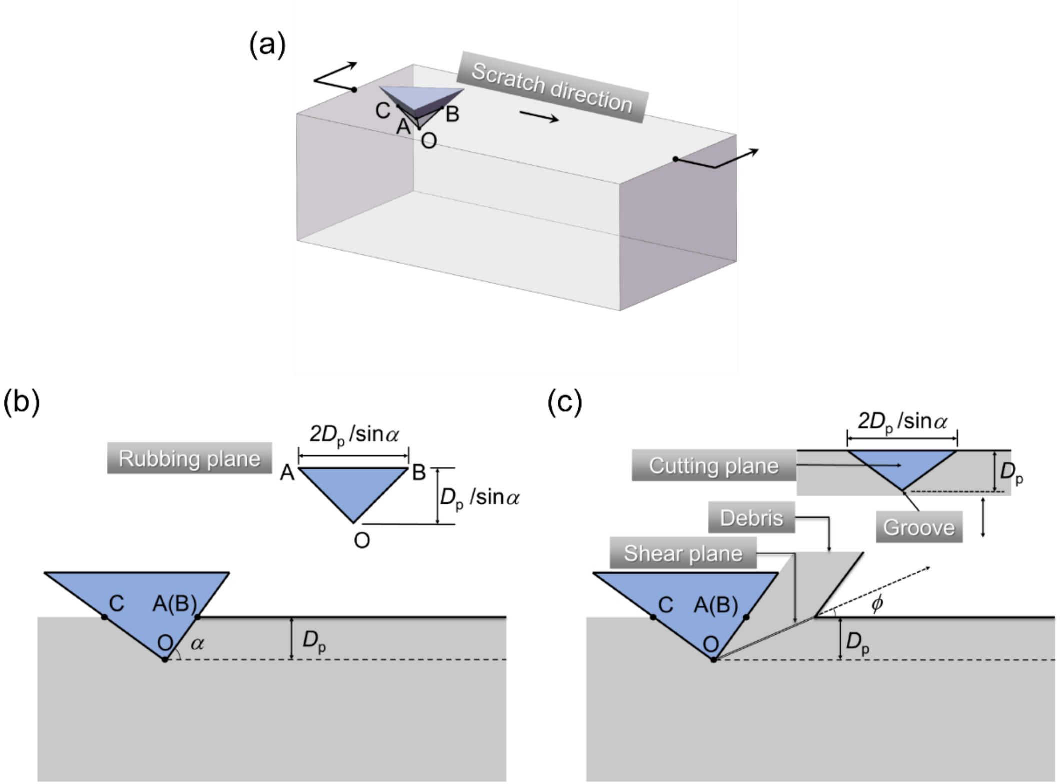

Figure S4. (a) 3D view of schematics of theoretical model. (b) Cross section view of ‘rubbing mode’ and (c) ‘cutting mode’.

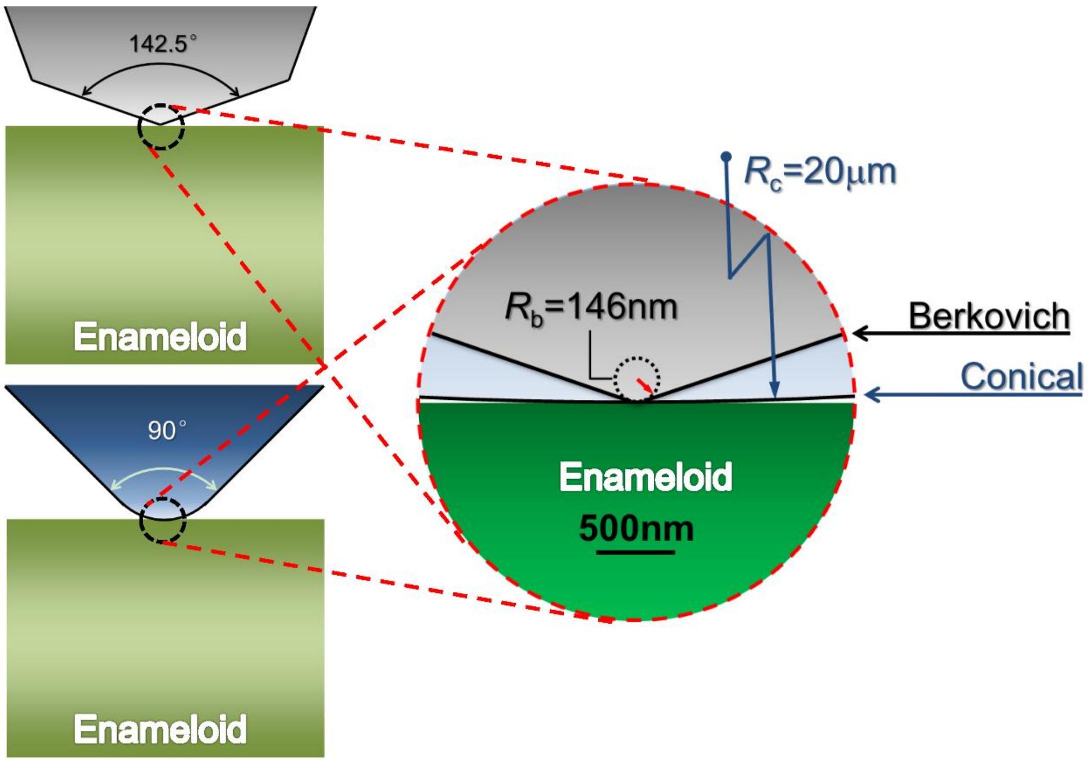

Figure S5. Comparison between Berkovich probe with radius of 146 nm and blunt conical probe (included angle = 90 °) with radius of 20 μm.

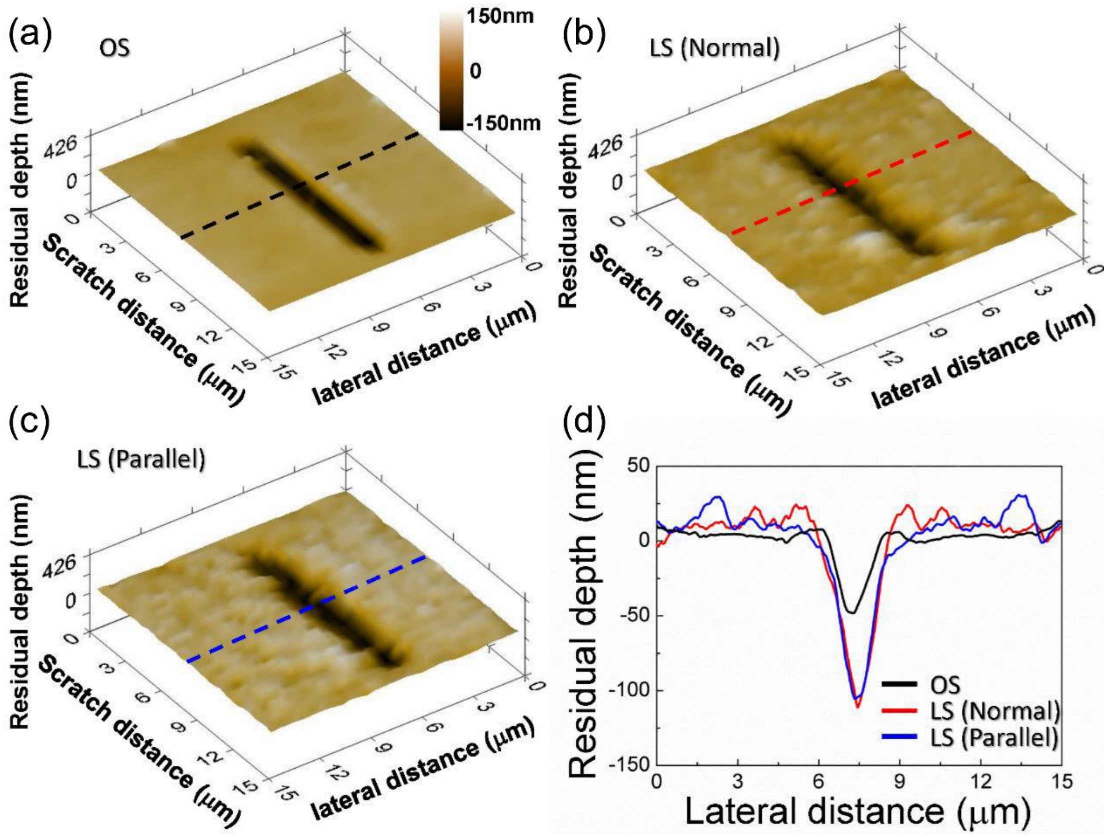

Figure S6. AFM images of grooves scratched by 90 °conical tip with radius of 20 μm and the cross-section profiles of the grooves. AFM images of scratches on the (a) OS; (b) LS with scratching direction is normal to the OS; (c) LS with scratching direction is parallel to the OS. (d) The cross-section profiles of the grooves at the position of dotted lines in (a), (b) and (c).

Table S1. The mechanical properties of HAp calculated from (001), (010), (110), and $(1\bar{1}0)$ planes in comparison with the values reported in literature.

Planes	τ_y (GPa)	H (GPa)	E_r (GPa)	γ_s (J/m^2)
(001)	3.92	8.84 (7.06[2,3])	160.9 (142.92[2,3])	1.32 (1.345[4],1.692[5],1.043[6])
(010)	3.08	8.38 (6.41[2,3])	151.2 (137.98[2,3])	1.20 (1.692[5],1.709[6])
(110)	2.92	8.51	127.4	1.33 (1.692[5])
$(1\bar{1}0)$	3.02	8.18	103.8	1.34 (1.692[5])
